# Morphological and Functional Changes of Corneal Nerves and Their Contribution to Peripheral and Central Sensory Abnormalities

**DOI:** 10.3389/fncel.2020.610342

**Published:** 2020-12-10

**Authors:** Adrian Guerrero-Moreno, Christophe Baudouin, Stéphane Melik Parsadaniantz, Annabelle Réaux-Le Goazigo

**Affiliations:** ^1^Sorbonne Université, INSERM, CNRS, Institut de la Vision, IHU FOReSIGHT, Paris, France; ^2^CHNO des Quinze-Vingts, IHU FOReSIGHT, INSERM-DGOS CIC 1423, Paris, France; ^3^Department of Ophthalmology, Ambroise Paré Hospital, AP-HP, University of Versailles Saint-Quentin-en-Yvelines, Boulogne-Billancourt, France

**Keywords:** cornea, trigeminal ganglion, pain, inflammation, peripheral and central sensitization

## Abstract

The cornea is the most densely innervated and sensitive tissue in the body. The cornea is exclusively innervated by C- and A-delta fibers, including mechano-nociceptors that are triggered by noxious mechanical stimulation, polymodal nociceptors that are excited by mechanical, chemical, and thermal stimuli, and cold thermoreceptors that are activated by cooling. Noxious stimulations activate corneal nociceptors whose cell bodies are located in the trigeminal ganglion (TG) and project central axons to the trigeminal brainstem sensory complex. Ocular pain, in particular, that driven by corneal nerves, is considered to be a core symptom of inflammatory and traumatic disorders of the ocular surface. Ocular surface injury affecting corneal nerves and leading to inflammatory responses can occur under multiple pathological conditions, such as chemical burn, persistent dry eye, and corneal neuropathic pain as well as after some ophthalmological surgical interventions such as photorefractive surgery. This review depicts the morphological and functional changes of corneal nerve terminals following corneal damage and dry eye disease (DED), both ocular surface conditions leading to sensory abnormalities. In addition, the recent fundamental and clinical findings of the importance of peripheral and central neuroimmune interactions in the development of corneal hypersensitivity are discussed. Next, the cellular and molecular changes of corneal neurons in the TG and central structures that are driven by corneal nerve abnormalities are presented. A better understanding of the corneal nerve abnormalities as well as neuroimmune interactions may contribute to the identification of a novel therapeutic targets for alleviating corneal pain.

## Introduction

**Figure 1:**
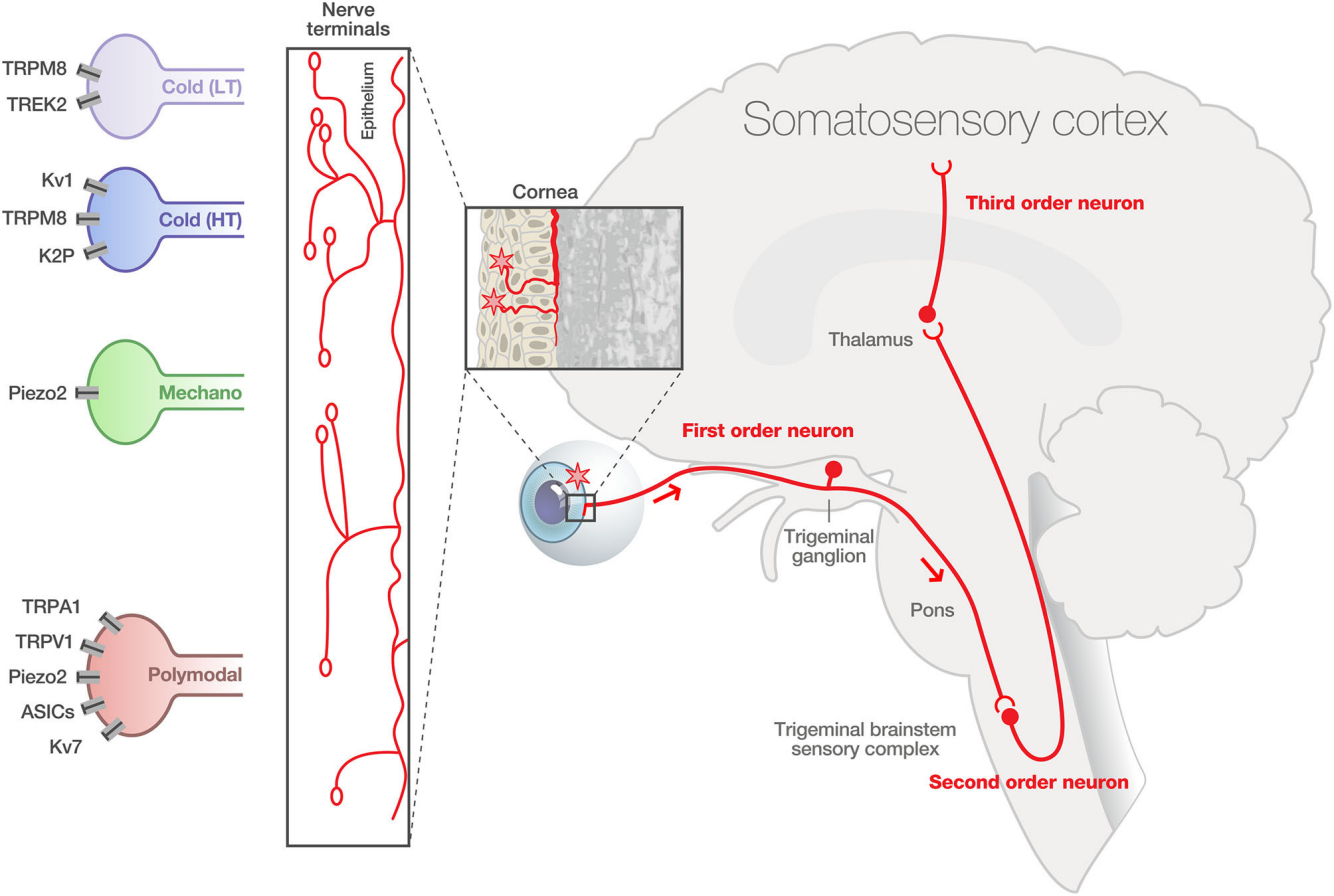
Physiological corneal pain processing. The cornea contained a high density and heterogeneity of A delta and C fibers expressing specialized transient receptor channels, able to respond to diverse environmental stimuli of varying intensities (cold, mechanical, and polymodal). The intraepithelial nerve fibers exhibit distinct morphologies: complex, ramified, and simple. First order sensory neurons innervating the cornea have their cell body in the ophthalmic branch (V1) of the trigeminal ganglion (TG). The central terminals of these pseudo unipolar neurons synapse in the trigeminal brainstem sensory complex with second order nociceptive projection neurons. These neurons project to the thalamus and synapse with third-order neurons projecting to cortical regions (primary somatosensory cortex). LT, low-threshold cold thermoreceptors; HT, high-threshold cold thermoreceptors. Modified from Belmonte et al. (2015) and Rosenthal and Borsook (2016).

The cornea is the most densely innervated tissue in the human body. Corneal innervation is estimated as 300–600 times and 20–40 more innervated than skin and tooth pulp (Muller et al., 2003; Marfurt et al., 2010). The human corneal innervation is organized into four layers: mid stromal nerves, subepithelial plexus, subbasal nerve plexus, and intraepithelial nerve terminals (Muller et al., 2003; Marfurt et al., 2019). The sensory innervation comes from corneal primary afferent neurons whose cell bodies are located in the ophthalmic (V1) branch of the trigeminal ganglion (TG). Corneal neurons represent only 1–5% of total trigeminal neurons (Marfurt and Del Toro, 1987; Launay et al., 2015). The central axons of corneal sensory neurons terminate in two regions of the spinal trigeminal complex (V or Sp5): the sensory trigeminal subnucleus interpolaris/caudalis (Vi/Vc) transition and the subnucleus caudalis/upper cervical cord (Vc/C1) junction regions (Marfurt and Del Toro, 1987; Strassman and Vos, 1993; Meng and Bereiter, 1996). The second order neurons project to the thalamus and synapse with third order neurons projecting to cortical regions (primary somatosensory cortex) (Figure 1). Therefore, any corneal injury or corneal nerve abnormalities may trigger molecular, cellular, and functional changes within the TG and the central nervous system, which may contribute to acute and chronic pain.

## Functional Classification of Corneal Fibers

The cornea receives sensitive and autonomous innervation. Corneal nerve fibers have been classified according to different criteria—morphology (intraepithelial nerves), conduction velocity (diameter, myelinated or not), and function/modality (stimulus sensitivity and specificity, electrophysiological properties)—and according to the expression of very specific biochemical markers. Unlike somatic nerve innervation, the cornea lacks A-beta fibers. Cornea is composed of two types of fibers:

- the high speed myelinated Aδ fibers with a larger diameter represent about 20% of the total population of corneal afferent fibers (MacIver and Tanelian, 1993a; Bron et al., 2014).
- slower-conducting unmyelinated C fibers with a small diameter are the most frequent (around 80%) (Gallar et al., 1993; MacIver and Tanelian, 1993a; Hirata and Meng, 2010).

Two sub-populations of C fibers have been identified: peptidergic and non-peptidergic fibers. In rodents, peptidergic fibers contain substance P (SP, 10–20%) or calcitonin gene-related peptide (CGRP, 40–60%). All SP^+^ terminals are CGRP^+^ in mice (Ivanusic et al., 2013). Other mediators are also found, such as neurokinin A, serotonin, somatostatin, as well as cholecystokinin or gastrin (Gonzalez-Coto et al., 2014). In humans, to date, only substance P and CGRP have been clearly identified in corneal fibers. A second population of C fibers, the non- peptidergic C fibers, does not express these peptides but has a strong affinity for isolectin B4. In mice, about 20% of corneal neurons are non-peptidergic (Ivanusic et al., 2013).

The majority of corneal nerve fibers are nociceptive and cold thermoreceptors fibers and the remaining fibers are sympathetic or parasympathetic post-ganglion fibers of the autonomic nervous system.

Autonomic nervous fibers of sympathetic (upper cervical ganglion) and parasympathetic system (ciliary ganglion) participate in the regulation of corneal wound healing and re-epithelialization (Jones and Marfurt, 1996; Xue et al., 2018; Xiao et al., 2019). Theses biological processes involve a number of concerted events including cell migration, proliferation, inflammation, and differentiation and extracellular matrix remodeling (Ljubimov and Saghizadeh, 2015). Interestingly, it was recently reported that the mouse autonomous system modulates inflammation and epithelial renewal after corneal abrasion through the activation of distinct local macrophages (Xue et al., 2018; Xiao et al., 2019).

In rodents and cats, these fibers represent about 10% of corneal fibers. Parasympathetic fibers, originating from the ciliary ganglion, contain intestinal vasoactive peptide (VIP), neuropeptide Y, galanin, and Met-enkephalin (Jones and Marfurt, 1998; Muller et al., 2003).

Electrophysiological studies showed that the cornea is innervated by three distinct classes of peripheral sensory nerve fibers:

**Mechano-nociceptors** (Aδ fibers) are activated only by soft mechanic forces in the order of magnitude closed to that required to damage corneal epithelial cells. In general, mechano-nociceptors only fire when a mechanical stimulus is applied or removed in a phasic way (Belmonte et al., 1991; MacIver and Tanelian, 1993b). Piezo2, a long transmembrane protein and non-selective cation channel mechanically activated (Coste et al., 2010; Shin et al., 2019), has emerged as a marker of mechano-nociceptors in primary sensory neurons.

**Cold receptors** (Aδ and C fibers) are activated by thermal changes (Gallar et al., 1993; Carr et al., 2003). These are classified into two subgroups (Alcalde et al., 2018).

° *High background-low threshold (HB-LT) cold receptors* are the principal generators of spontaneous activity (high background) at ocular surface constant temperature. These fibers change their firing frequency according to slight temperature changes (1–2°C, low threshold). Additionally, they detect changes in ocular surface osmolarity (Hirata and Meng, 2010; Parra et al., 2014; Gonzalez-Gonzalez et al., 2017). A hyperosmolar tear film with values close to those found in dry eye patients increases the sensitivity of cold-sensitive neurons.
° *Low background-high threshold (LB-HT) cold receptors* do not contribute as HB-LT to basal ongoing activity (low background), but instead fire under sharp drops in temperature (>4°C) (Gonzalez-Gonzalez et al., 2017).

TRPM8 (transient receptor potential cation channel subfamily M member 8) is considered as a marker of cold receptors. TRPM8 is activated by menthol and by cold (<23°C) (McKemy et al., 2002; Peier et al., 2002). Due to these characteristics, it is the putative principal sensor of cold.

Indeed, TRPM8 receptors appear to be first activated on the ocular surface after evaporation of the tear film (Belmonte et al., 2017) and mild cooling of the ocular surface has been reported to increase lacrimation via TRPM8 activation of corneal cool primary afferent neurons (Robbins et al., 2012). In the same line, it was found that TRPM8 knockout mice have a lower level of basal tear flow (Parra et al., 2010).

**Polymodal nociceptors** (Aδ and C fibers) are the most abundant population in the cornea (Gonzalez-Gonzalez et al., 2017). These fibers are activated by different types of stimuli:

° *Mechanical:* In contrast to pure mechano-nociceptors, polymodal nociceptors have a lower threshold and fire continuously (tonic) while the stimulus is present (Gallar et al., 1993).
° *Thermal*: They are activated by temperatures above 37°C (Gallar et al., 1993).
° *Chemical*: There is a broad variety of molecules activating these nociceptors, including protons (pH drops) and inflammatory mediators (prostaglandins, bradykinin, and capsaicin) (Chen et al., 1997).

TRPV1 (transient receptor potential cation channel subfamily V member 1), a non-selective cation channel, is considered as a marker of polymodal sensory fibers (Belmonte et al., 1991). Thus, it represents the principal sensor of hot painful stimulus.

Retrograde tracing anatomical experiments were used to identify the whole population of corneal neurons in the TG and to determine the proportion of the corneal neurons expressing the molecular marker of polymodal, mechanoreceptors and cold cells. This studies have demonstrated by *in situ* hybridization that Piezo2 represents 28–30% of corneal afferent neurons in guinea pig TGs and do not co-express TRPV1 (Alamri et al., 2015) or TRPM8 (Bron et al., 2014). The co-expression of TRPM8 and TRPV1 occurs in 6% of TRPV1^+^ cells corresponding to 31% of TRPM8^+^ cells (Alamri et al., 2015). Interestingly, all SP-reactive neurons were also TRPV1^+^ in rats (Murata and Masuko, 2006). Therefore, they constitute separate populations of corneal sensory neurons. The proportion of corneal neurons expressing TRPV1 varies depending on the animal species and the tracer used: in rats, 37% when traced with cholera toxin (Murata and Masuko, 2006) and 23% when traced with Fluoro-Gold (Nakamura et al., 2007); in guinea pigs, it reaches 45% of corneal neurons when traced with Fast blue (Alamri et al., 2015). TRPM8 is expressed in 8–15% of corneal neurons in guinea pigs (Bron et al., 2014; Alamri et al., 2015) and 18–22% in mice (Ivanusic et al., 2013; Alcalde et al., 2018).

Anatomical studies focusing on corneal nerve terminals found that corneal polymodal (TRPV1^+^) and cold (TRPM8^+^) neurons have distinct nerve terminal morphologies. It was demonstrated that, compared to TRPV1 neurons, TRPM8-IR corneal nerve endings in both guinea pig and mouse corneal epithelia had complex (longer and more branched) morphology (Ivanusic et al., 2013; Alamri et al., 2015, 2018; He et al., 2019) (Figure 1). The presence of Piezo2^+^ channels has been recently found in corneal nerve fibers in mice (Fernandez-Trillo et al., 2020).

Accumulating fundamental and clinical studies have reported morphological, structural, and/or functional corneal nerve abnormalities, which may contribute to pain-related sensory abnormalities.

## Nociception, Pain, and Neurosensory Abnormalities

Nociception is the neural process of encoding noxious stimuli, whereas pain is defined as *an unpleasant sensory and emotional experience associated with, or resembling that associated with, actual or potential tissue damage* [the International Association for the Study of Pain (IASP)] (Raja et al., 2020).

Nociceptive pain is a physiological protective warning signal against imminent or ongoing tissue damage. Nociceptive pain starts at the periphery by the activation of nociceptors by physical tissue destruction or by chemical exposure, or thermal processes. Inflammatory pain occurs in the presence of tissue injury and active inflammation. It is the consequence of the activation and alteration of nociceptor function both by immune signals and signals arising from damaged cells (Woolf, 2020).

In this context, proinflammatory mediators participate in the sensitization of the nerves, meaning the reduction of the pain threshold (allodynia) and exacerbated response to a painful stimulus (hyperalgesia). Therefore, sensitization can occur both in the periphery (peripheral sensitization) and in the central nervous system (central sensitization).

Pain can extend beyond its protective usefulness, lasting in the absence of nociceptive stimuli. Neuropathic pain, a chronic pain condition, is defined by the IASP as “*pain caused by a lesion or disease of the somatosensory nervous system*.” By definition, the origin of the neuropathic pain can be peripheral or central. There is important interest in the field, as the current treatments for alleviating neuropathic pain are often inefficacious and/or produce severe side effects.

In the ocular surface, and more specifically in the cornea, all of these physiological and pathophysiological conditions can occur. Pain can manifest as a result of a noxious stimulus or damage in the ocular surface anatomy (nociceptive pain), or it can result from abnormalities in the ocular surface neurosensory apparatus itself (neuropathic pain).

Neurosensory abnormalities within the corneal nociceptive pathway could lead to pathological conditions such as pain and ocular discomfort that are hallmark of dry eye disease (DED) and the emerging ocular neuropathic pain. Corneal neuropathic pain may have various etiological origins, which include DED, persistent corneal nerve injury, and photorefractive surgeries (Dieckmann et al., 2017; McMonnies, 2017; Aggarwal et al., 2019). Dry eye is defined as a “*multifactorial disease of the ocular surface characterized by a loss of homeostasis of the tear film, and accompanied by ocular symptoms, in which tear film instability and hyperosmolarity, ocular surface inflammation and damage, and neurosensory abnormalities play etiological roles"* (Craig et al., 2017). The prevalence of DED, which ranges from 5 to 50% of the adult population, increases with age, especially after 50 years, and affects more women than men (Stapleton et al., 2017). DED has gained recognition as a public health problem given its prevalence, morbidity, and cost implications.

Importantly, the cellular and molecular mechanisms underlying the neurosensory abnormalities observed after corneal damage and in DED are currently an open and very active research field.

This increasing understanding of the corneal nerve morphological and functional aspects will bring new insights into their contribution to the physiology and pathophysiology of corneal pain perception and will provide novel opportunities for more efficient therapeutic treatments for this ocular surface diseases.

In the context of other ocular surface diseases, excellent reviews have already provided detailed information about the morphological and functional abnormalities of corneal nerves in herpetic keratitis, neuropathic keratitis, or keratoconus (Cruzat et al., 2017) and diabetes (Markoulli et al., 2018).

The purpose of this review is first to provide an overview of the morphological, molecular, and cellular changes of corneal nerves in rodents after corneal damage and persistent DED. The cellular and molecular changes of corneal neurons in the TG driven by corneal nerve abnormalities are discussed next. Moreover, we depict how peripheral neuroimmune interactions shape the peripheral and the central nervous system. Clinical evidence for corneal nerve abnormalities associated with DED and corneal neuropathic pain is also presented as well as several important questions that remain to be addressed.

## Fundamental Studies on Morphologic Abnormalities of Corneal Nerve Terminals

The cornea is a valuable tissue for studying peripheral sensory nerve morphology and function due to its transparency, dense innervation, and accessibility. Corneal nerve structure and function are adversely affected by many ophthalmic and systemic disorders. Experimental descriptive studies exploring the changes of corneal nerve morphology have improved our understanding of how corneal nerve density is altered under DED and corneal injury conditions. Thus, transgenic thy1-yellow fluorescent protein (thy1-YFP) mice, in which corneal nerves express the YFP protein driven by thy1 promoter, represent a useful model for *in vivo* investigation of peripheral nerve structure (Yu and Rosenblatt, 2007; Namavari et al., 2011; Chaudhary et al., 2012; Sarkar et al., 2012, 2019; Bouheraoua et al., 2019). A decrease in stromal nerve fiber density as well as inflammation were observed in corneas from thy1-YFP mice submitted to experimental DED induced by chronic topical instillation with benzalkonium chloride, a quaternary ammonium used as preservative (Sarkar et al., 2012).

In addition, immunohistochemistry detecting all corneal nerve fibers in tissue samples and *in vivo* confocal microscopy (IVCM, a non-invasive high-resolution real-time imaging device allowing layer-by-layer analysis of the corneal ultrastructure) have covered aspects of the morphological basis of corneal nerve changes in rodents both in physiological and pathophysiological conditions. A decrease in corneal nerve density was reported in preclinical models of experimental DED induced by prolonged (28 days) (Simsek et al., 2019) or acute (up to 10 days) (Stepp et al., 2018a) scopolamine administration. Similar observations were made in cd25 null mice, which constitute a model of Sjögren Syndrome dry eye (Stepp et al., 2018b).

Tear hyperosmolarity plays a critical role in the initiation and/or perpetuation of DED, which could have consequences with respect to corneal nerve abnormality. Indeed, a reduction in the density of corneal intraepithelial nerves and terminals, in addition to a sensitized ocular surface to hypertonicity, was also recently reported in a murine model of tear hyperosmolarity (Guzman et al., 2020).

Furthermore, surgically induced chronic dry eye models obtained after the unilateral removal of extra-orbital lacrimal gland in mice (Yamazaki et al., 2017) or after the excision of the extraorbital lachrymal gland and Harderian gland had similar findings (Fakih et al., 2019): a reduction in corneal nerve density accompanied with corneal allodynia. Finally, structural abnormalities including a profound loss of nerve density in the sub-basal nerves and increasing of beading and tortuosity of stromal nerve trunks also occurred in a model of corneal injury-induced neuropathic pain (Cho et al., 2019; Pham and Bazan, 2020).

Altogether, these morphological changes in corneal density innervation reported from the fundamental studies above resemble those observed in patients with corneal injury, patients who underwent refractive surgery, and patients with DED and ocular neuropathic pain (see paragraph dedicated to clinical data). One key initiative is to determine the precise underlying molecular mechanisms responsible for these morphological changes.

Recent advances in tissue clearing methods and light-sheet fluorescence microscopy have provided unprecedented access to structural and molecular information from intact tissues. A 3D high resolution imaging of an intact eyeball using a tissue clearing system derived from CLARITY and advanced light sheet microscopy in wild type fluorescently labeled transgenic mice of Prox-1-GFP or Thy1-YFP (Yang et al., 2020) was recently reported. The technology, applicable to various mouse strains (wild type or fluorescently labeled), and a spectrum of ocular components and cell types (i.e., corneal nerves, blood vessels, immune cells.) represent multiple opportunities for further architectural/morphological studies in basal and pathological ocular conditions.

## Evidence for Functional Abnormalities of Corneal Nerve Terminals in Experimental Models of Corneal Injury and DED

Corneal nerve dysfunction has been reported in multiple ocular surface conditions including DED, herpetic keratitis, and after surgery (see review McKay et al., 2019). Peripheral sensitization is defined as increased responsiveness and a reduced threshold of nociceptive neurons in the periphery of the stimulation. Such phenomenon can occur in the cornea (see reviews Belmonte et al., 2004, 2017). Indeed, chronic peripheral nerve injury and local inflammation are known to participate in the development of peripheral sensitization. Immune cells locally release cytokines, chemokines, lipids, and growth factors that act on peripheral nociceptors. In turn, nociceptors actively release neuropeptides from their peripheral nerve terminals that modulate the activity of innate and adaptive immune cells (Chiu et al., 2012; Baral et al., 2019).

The development of ocular pain after corneal damage or persistent DED is believed to be due to the abnormal hyperexcitability (sensitization) of corneal nerve terminals. Single corneal nerve terminals and ciliary nerve fiber activity recordings are the two main experimental approaches currently reported to evaluate the functional changes of ongoing or evoked nerve activity in fundamental studies. Single unit extracellular recordings to evaluate polymodal and cold-sensitive corneal nerve terminals have been described using *ex vivo* isolated corneas in mice and in guinea pigs (Acosta et al., 2013, 2014; Kovacs et al., 2016b; Gonzalez-Gonzalez et al., 2017; Hirata et al., 2017; Alamri et al., 2018; Pina et al., 2019).

Extracellular recordings of ciliary nerve fiber activity have been described in whole eye preparations *in vivo* in cats (Gallar et al., 1993; Acosta et al., 2001, 2007) and *ex vivo* in isolated guinea pigs (McLaughlin et al., 2010; Acosta et al., 2013, 2014), rabbits (Beuerman et al., 1992), and mice (Fakih et al., 2019; Joubert et al., 2019). *Ex vivo* extracellular recordings of ciliary nerve activity being performed in double compartment chambers offer the possibility of applying chemical, mechanical, and thermal stimulations directly to the cornea to specifically study the responses of polymodal nociceptors, mechano-nociceptors, and cold thermoreceptors. By using this approach, a recent study showed a correlation between corneal hypersensitivity (decreased mechanical threshold), higher spontaneous ciliary nerve fiber activity, and the higher responsiveness of corneal polymodal nerve fibers from mice submitted to an acute corneal nerve injury associated with local inflammation (Joubert et al., 2019). The abnormal responses of the polymodal nociceptors (lower activation and decreased latency of the impulse discharge) observed may be a consequence of inflammation, known to play a major role in corneal nerve sensitization (McMahon and Wood, 2006; Gallar et al., 2007; Parra et al., 2014).

**Figure 2:**
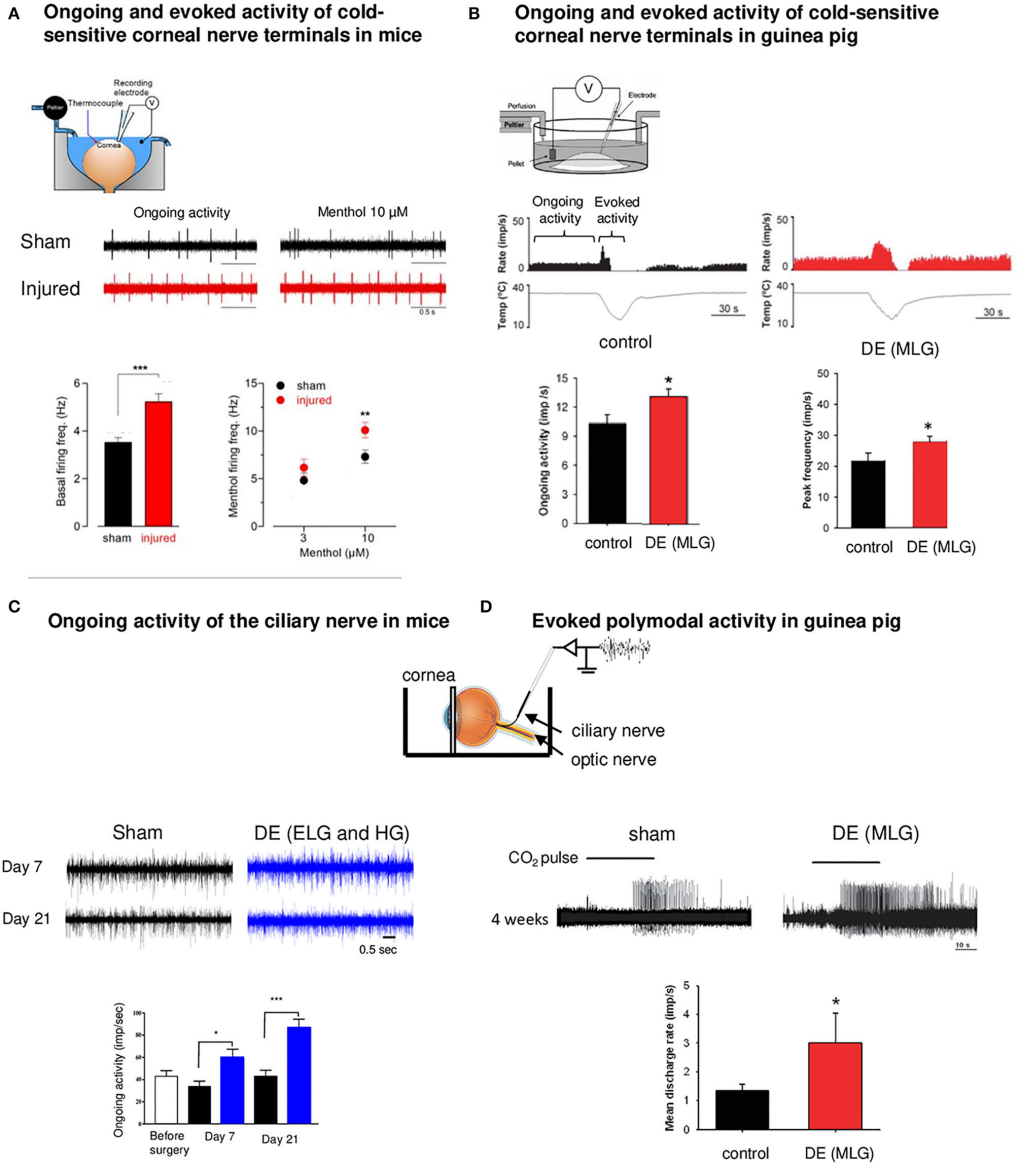
Functional abnormalities of corneal nerves following corneal damage and DE condition. *Ex vivo* electrophysiological recordings of corneal nerves in models of corneal nerve damage and DE conditions. **(A)** Electrophysiological set-up. Spontaneous (ongoing) and evoked (menthol 10 μM) activity of cold sensitive corneal nerve terminals in a mouse model of corneal nerve injury (red traces) relative to control (sham animals, black traces). Note the increase in corneal nerve activity for both conditions in the injured cornea. Data plotted as mean ± SEM. *P < 0.05, **P < 0.01, ***P < 0.001. **(B)** Electrophysiological set-up of ongoing and evoked activity of cold nerve terminals in a guinea pig model of DE obtained after the main lachrymal gland (MLG) excision. Ongoing activity and peak frequency evoked by cooling-ramp were increased 4 weeks after MLG excision. **(C)** Electrophysiological set-up for extracellular recording of the ciliary nerve. Ongoing ciliary nerve activity 7 days and 21 days after the excision of extraorbital (ELG) and Harderian gland (HG) in mice (blue traces) relative to sham animals (black traces). In both conditions, we noted higher ongoing (spontaneous) firing frequency in DE animals. **(D)** Activity of polymodal nerve fibers activated by 30 s CO_2_ pulse in control (sham) and MLG guinea pigs 4 weeks after surgery. The mean discharge rate during the CO_2_ pulse was higher after the surgery compared to sham. Note the higher responsiveness of polymodal nerve fibers (decreased latency) in DE animals. Modified from Acosta et al. (2013), Kovacs et al. (2016b), Fakih et al. (2019), and Pina et al. (2019).

Another category of corneal nerve fiber changes their modalities after corneal injury and DED: cold TRPM8 corneal fibers. The contribution of TRPM8 channels to cold transduction in peripheral nerve terminals was confirmed in TRPM8-KO mice. KO animals do not show a response to noxious cold stimulus (Madrid et al., 2006). In lacrimo-deficient guinea pigs, cold nerve terminals exhibited enhanced spontaneous activity (hyperexcitability) and cold response in addition to a reduced cold threshold to cooling ramps compared to nerve terminals recorded from control animals (Kovacs et al., 2016a,b) (Figure 2).

The changes in corneal cold thermoreceptor firing were also observed experimentally after a peripheral corneal axotomy (Pina et al., 2019). Corneal injury evoked electrophysiological alterations in TRPM8 corneal neurons, which manifest by an enhanced sensitivity of the corneal TG neurons to cold (Pina et al., 2019). The application of menthol (10 μM), a TRPM8 agonist, induced a more pronounced increase in the firing rate in corneas of injured mice than in those of sham animals (Pina et al., 2019) (Figure 2).

Furthermore, a study reported that tear fluid hyperosmolarity (325–1,005 mOsm·kg-1), recognized as an important pathogenic factor in dry eye syndrome, increases nerve impulse activity of cold thermoreceptor endings of the cornea (Parra et al., 2014).

Thus, abnormal activity and the responsiveness of peripheral corneal cold thermoreceptors underlie the unpleasant sensations experienced by patients with DED.

In line with these observations, a severe DED induced by the excision of extraorbital lachrymal and Harderian glands in mice provoked corneal mechanical allodynia and corneal inflammation associated with an increase in the ongoing ciliary nerve fiber electrical activity compared to control mice (Fakih et al., 2019) (Figure 2). In addition, during photorefractive keratectomy, another corneal injury condition, functional and morphologic alterations in mechanical, polymodal, and cold sensory nerve fibers of the cornea have been noted, which can lead to postoperative pain (Bech et al., 2018).

In preclinical models of other corneal and conjunctival disorders characterized by an important inflammatory component, such as allergic keratoconjunctivitis (Acosta et al., 2013) or UV Keratitis (Acosta et al., 2014), an inhibition of cold receptors and sensitization of polymodal nociceptors were reported. These results contrast with the preclinical and clinical data from DED condition, in which both populations of corneal fibers (polymodal and thermoreceptors) showed sensitization (abnormal responsiveness and spontaneous firing). These aspects have been recently reviewed (Belmonte, 2019).

Nociceptive nerve terminals contain specific elements for detecting, transmitting, and modulating noxious signals (Waxman, 1999). Among them, the spike initiation zone located in the axon initial segment (AIS) corresponds to the site where action potentials are initiated and represents a critical element in neuronal excitability (Yamada and Kuba, 2016). Structural properties, such as the location relative to the soma and the length of the AIS, can change in an activity-dependent manner that can fine-tune the neuronal output properties (Jones and Svitkina, 2016; Yamazaki et al., 2017). A recent study has not only identified the precise location of the NaV-dependent spike initiation zone in nociceptive corneal nerve terminals *in vivo*, but has also demonstrated a plasticity in the spike initiation zone (Goldstein et al., 2019). Corneal inflammation shifts the Nav-spike initiation zone toward the terminal end, rendering it hyperexcitable in a model of inflammation-induced peripheral hyperalgesia (Goldstein et al., 2019).

Taken together, corneal nerve damage and inflammation trigger corneal sensory nerve dysfunctions (mostly sensitization); these peripheral nerve abnormalities may account for ocular pain syndromes. The above studies also reinforce the importance of neuroimmune interactions that participate in corneal hypersensitivity, inflammation, and spontaneous and evoked corneal nerve fiber hyperactivity.

Nociceptors and immune cells interact bidirectionally, a communication made possible by the wide spectrum of cells involved and by the receptors and ligands they express (Jain et al., 2020). From these multiple interactions has emerged the notion of neuroimmune interactome, a comprehensive map of the bidirectional ligand-receptor “interactome” between sensory neurons and immune cells (Jain et al., 2020). In the future, peripheral neuroimmune interactome could be constructed during pathological conditions such as corneal inflammation and corneal pain conditions. Enhancing our knowledge of the nociceptor and immune cell communication as well as the plasticity of both actors may contribute to the identification of novel therapeutic targets.

## Molecular and Functional Changes in Trigeminal Corneal Neurons After Corneal Injury/Under Pathological Conditions

After peripheral nerve injury, primary sensory neurons show molecular and functional changes, resulting in hyperactivity and hyperexcitability (Berta et al., 2017). These maladaptive changes have been well-documented in dorsal root ganglion neurons in various inflammatory and pain models, but less is known about the possible alterations in the corneal nociceptive trigeminal pathways following ocular surface damage.

Anatomical studies using retrograde-labeled corneal neuron experiments have found a higher expression of TRPV1 in TRPM8^+^ cold-sensing corneal neurons in the TG in a model of DED, suggesting an enhanced responsiveness of TRPM8^+^ cells and leading to a cold allodynia (Hatta et al., 2019; Li et al., 2019). Moreover, single cell RT-PCR indicated that all TRPM8^+^/TRPV1+neurons express substance P (Tac1), while fewer TRPM8^+^/TRPV1- neurons express Tac1. It was proposed that TRPV1-dependent neuronal sensitization facilitates the release of the neuropeptide substance P from TRPM8^+^ cold-sensing neurons to signal nociception in response to cold (Li et al., 2019). However, such results are in contradiction with a retrograde tracing study which found that SP and TRPM8 were expressed in different TG neurons, suggesting that TRPM8^+^ corneal cells are non-peptidergic (He et al., 2019).

Several studies have reported changes in mRNA expression in TG following corneal damage. For example, abnormal SP and TRPM8 gene expression was found associated with corneal hypersensitivity in a model of corneal surgery (epithelium removal and one-third of the anterior stroma with a 2 mm trephine). These molecular and cellular changes occurring in the TG may contribute to the pathogenesis of corneal surgery-induced chronic pain (He et al., 2019). In a model of corneal alkali-burn injury, the increased gene expression of pro-inflammatory cytokines, tumor necrosis factor (TNF alpha), interleukin (IL)-6, SP, and its receptor NK1 (NK1) were reported in the ipsilateral TG (Ferrari et al., 2014). Changes in mRNA expression were also found in TG from an experimental model of DED obtained after 7 days of topical benzalkonium chloride (BAK). This model developed corneal inflammation and corneal hypersensitivity, and an increase in the gene expression of pro-inflammatory cytokines (IL-6 and TNF-alpha) was reported (Launay et al., 2016). Additionally, neuronal activation (FOS), neuronal injury (ATF3), astrocyte (GFAP), and oxidative (INOS2 and NOX4) markers were found to be increased in the ipsilateral TG from animals submitted to a chronic DED characterized by corneal nerve damage, inflammation, and corneal hypersensitivity (Fakih et al., 2019).

Growing evidence indicates that both CGRP and SP play a key role in the development of peripheral sensitization and are implicated in the development of neurogenic inflammation (Chiu et al., 2012), giving a rational to all changes previously described. In this line, SP has a robust effect on monocytes and macrophages and triggers their release of pro-inflammatory cytokines, including IL-1, TNF, and IL-6, via ERK/p38 MAPK-mediated NF-κB activation. Interestingly, both neuropeptides have been found to be abundant in corneal neurons, and a transcriptome signature in the TG using unbiased RNA sequencing revealed an upregulation of tachykinin precursor 1 (*Tac1*) that encodes SP and Calcb, which encodes CGRP after corneal injury (Pham et al., 2020).

Monitoring c-Fos, ATF3, and c Jun immunopositive cells is a commonly used approach to studying the activation and damage of primary sensory neurons from dorsal root and trigeminal ganglia. cFos, c-Jun, and ATF3 protein expressions were shown to be increased in the ipsilateral TG from animals with corneal damage (De Felipe and Belmonte, 1999; Launay et al., 2016; Fakih et al., 2019; Reaux-Le Goazigo et al., 2019). Both CGRP and ATF3 protein expressions in TG cell bodies increased after injury and returned to a normal level by 1 week, paralleling the time course of changes in nociceptive responses (Hegarty et al., 2018). These increased expressions of FOS, CGRP, and ATF3 in primary sensory neurons may contribute to the activation of central pain pathways in response to sustained ocular stimulation, leading to the centralization of pain (Levine et al., 1993).

Under local inflammation or after tissue damage, cytokines, prostaglandins, nerve growth factor, and bradykinin signals increase TRPV1 expression and/or TRPV1 activity in sensory neurons (Pinho-Ribeiro et al., 2017). In the context of DED, TRPV1 protein levels have been found to increase in the ipsilateral TG, and this TRPV1 upregulation was associated with enhanced eye wipe behavior after hypertonic saline and capsaicin instillation in a rat model for aqueous tear-deficient DE (Bereiter et al., 2018).

In the same way, it has been observed that capsazepine, a TRPV-1 antagonist, prevented dry eye sensitization of cool cells to capsaicin (Hatta et al., 2019) and reduced polymodal responsiveness to acidic stimulation in an allergic eye model (Acosta et al., 2013). Moreover, TRPV-1 pharmacological blockade decreases SP release in cold allodynia (Li et al., 2019).

Neurons in sensory dorsal and trigeminal ganglia are surrounded by satellite glial cells (SGCs) (Hanani, 2005). Activation of SGCs is characterized by GFAP upregulation in the injured trigeminal nerve branch associated with the development of hyperalgesia (Vit et al., 2006; Katagiri et al., 2012), but this has not been observed under non-pathological conditions (Shinoda et al., 2019). Interestingly, increased spontaneous pain behavior and corneal allodynia have been associated with morphological changes (hypertrophy) and upregulation of GFAP protein expression in the SGCs at the level of the ipsilateral trigeminal nerve V1 branch in preclinical models of corneal injury (Launay et al., 2016; Fakih et al., 2019; Reaux-Le Goazigo et al., 2019).

In addition, accumulative evidence reports that immune cells as monocytes/macrophages infiltrate into the TG and become activated, following orofacial pathologies, including peripheral trigeminal nerve trauma and orofacial inflammation (Iwata and Shinoda, 2019). Fundamental studies performed in mice reported that corneal injury induced an increase and activated resident and proliferated macrophages in mouse TGs (Ferrari et al., 2014; Launay et al., 2016; Fakih et al., 2019; Reaux-Le Goazigo et al., 2019). The infiltrated monocytes/macrophages show larger soma and thicker ramifications; such morphological structural changes indicate their activation (Shinoda et al., 2019). The above observations suggest that immune cells and resident glia in the TG could play a significant role in the modulation of ocular pain. The specific role of both the population of cells should be better investigated in the future in the context of ocular pain.

Though RTqPCR and immunohistochemistry have provided some important information about neuronal, glial activations and cellular morphological abnormalities following corneal pain conditions, the major limitation of these techniques is that they lack the ability to monitor the dynamic of these neuronal changes. Functional studies have been performed in trigeminal neurons to better understand their activation and how various corneal nerve injuries may alter their modalities. *In vivo* electrophysiology works have assessed action potentials from single neurons and/or clusters of neurons in rodent TGs (Lopez de Armentia et al., 2000; Veiga Moreira et al., 2007; Hirata and Meng, 2010; Kurose and Meng, 2013; Hirata et al., 2015; Quallo et al., 2015) under normal and pathological conditions. The literature is not as rich as the one from DRG neurons, but it provides important information about changes in the neuronal properties of corneal neurons under physiological and DE conditions.

Understanding the effect of ocular surface damage on corneal nociceptive neurons is crucial for developing a potential antalgic treatment against corneal pain. To this aim, several studies based on single unit recordings of corneal nociceptive neurons located in the TG have been assessed in preclinical models of DED and corneal injury to provide valuable insight into the normal and pathologic response of the corneal neurons. These studies highlighted the relationship between the electrophysiological signature (both fiber conduction velocity and electrical properties) and the altered responsiveness to various sensory modalities (chemical, thermal, and mechanical) of afferents innervating the cornea.

Thus, *in vivo* extracellular electrophysiological recordings performed in rat TG single neurons that innervated the cornea, before, and up to 3 h after, the ocular application of continuous hyperosmolar tears demonstrated that dry responses of corneal dry-sensitive neurons were depressed or even completely abolished by the hyperosmolarity of tears (Hirata et al., 2015).

**Figure 3:**
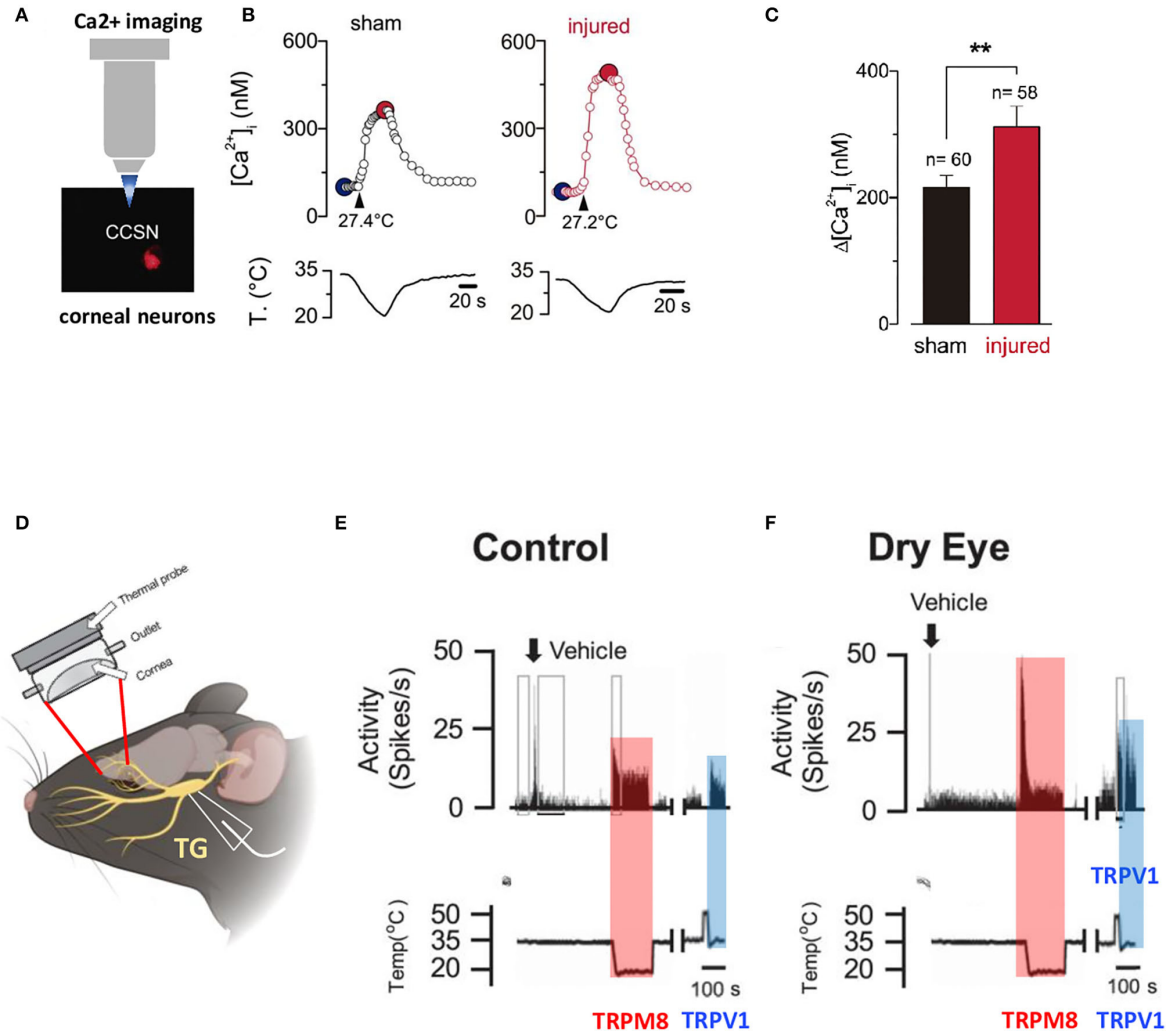
Altered responsiveness of corneal neurons following peripheral nerve damage and after lacrimal gland excision. **(A)** Altered cold sensitivity of corneal neurons induced by peripheral nerve damage. Calcium imaging on FM 1–43 labeled corneal neurons in culture. **(B)** Cold-evoked [Ca^2+^]i response to a temperature ramp of two representative corneal neurons from sham (black trace) and injured (red trace) mice. Large dots indicate the basal (blue) and maximal (red) [Ca^2+^]i during the cold stimulus. **(C)** Bar graphs of the mean amplitude of [Ca^2+^]i rises evoked by cold stimulation in the corneal neurons from sham and injured mice. Modified from Pina et al. (2019). Data plotted as mean ± SEM. **p = 0.0014. **(D)** Illustration of the custom-made chamber designed with inflow and outflow portals for maintaining a constant environment on the surface of the cornea while recording from neuronal cell bodies located in the TG. **(E,F)** Effects of vehicle and cool and heat-evoked activity in control **(E)** and lacrimal gland excision (LGE) animals **(F)**. Neuronal activity recorded from control animals and animals 2 weeks after LGE, following vehicle, cold stimulation (TRPM8 activation), and 3 μM capsaicin (TRPV1 activation) applications to the cornea. Copyright © 2019 the American Physiological Society (Hatta et al., 2019).

In a moderate dry eye experimental model obtained after unilateral extraorbital lacrimal gland excision, Kurose et al. demonstrated by a single unit extracellular recording of trigeminal neurons (8–10 weeks after the surgery) that dry eye sensitized corneal cool cells to the TRPM8 agonist menthol and to cool stimulation (Kurose and Meng, 2013). In the same experimental model, Hatta et al. showed that dry eye increased responsiveness to noxious heat and activation by capsaicin through TRPV1 (Hatta et al., 2019) (Figure 3). Pina et al. further demonstrated, in a mouse model of corneal injury, changes in cold sensitivity of corneal neurons in the TG through an enhanced functional expression of TRPM8 channels, suggesting that the increase in ocular dryness sensation and basal tearing rate observed after refractive surgery could be related to a disturbance in the TRPM8 responsiveness of cold sensory neurons (Kovacs et al., 2016b; Pina et al., 2019) (Figure 3).

## Evidence that Corneal Nerve Abnormalities Shape the Central Nervous System in Rodents

Corneal nociceptors, like other primary somatosensory neurons, are pseudo unipolar. They send a peripheral axon to innervate the corneal and a central axon to synapse on second-order neurons at two different locations of the brain stem nuclear complex: the Vi/Vc (trigeminal subnucleus interpolaris/caudalis transition region) and Vc/C1 (caudalis/upper cervical cord junction) areas of the trigeminal subnucleus caudalis region (Figure 1). Persistent ongoing activity in primary nociceptors may generate extensive changes in central pain processing-related structures, leading to maladaptive neuroplasticity. In addition, proinflammatory mediators participate not only in the sensitization of peripheral nerve terminals but also in the transfer of nociceptive information from the periphery to the central nervous system (Grace et al., 2014; Melik Parsadaniantz et al., 2015). More specifically, activated glial cells, which produce various proinflammatory cytokines (TNF alpha, IL-6, IL1 beta), neurotrophic factors, and chemokines, contribute to neuronal excitability and a central sensitization mechanism under trigeminal pain states (Iwata et al., 2011, 2017; Melik Parsadaniantz et al., 2015; Goto et al., 2016).

**Figure 4:**
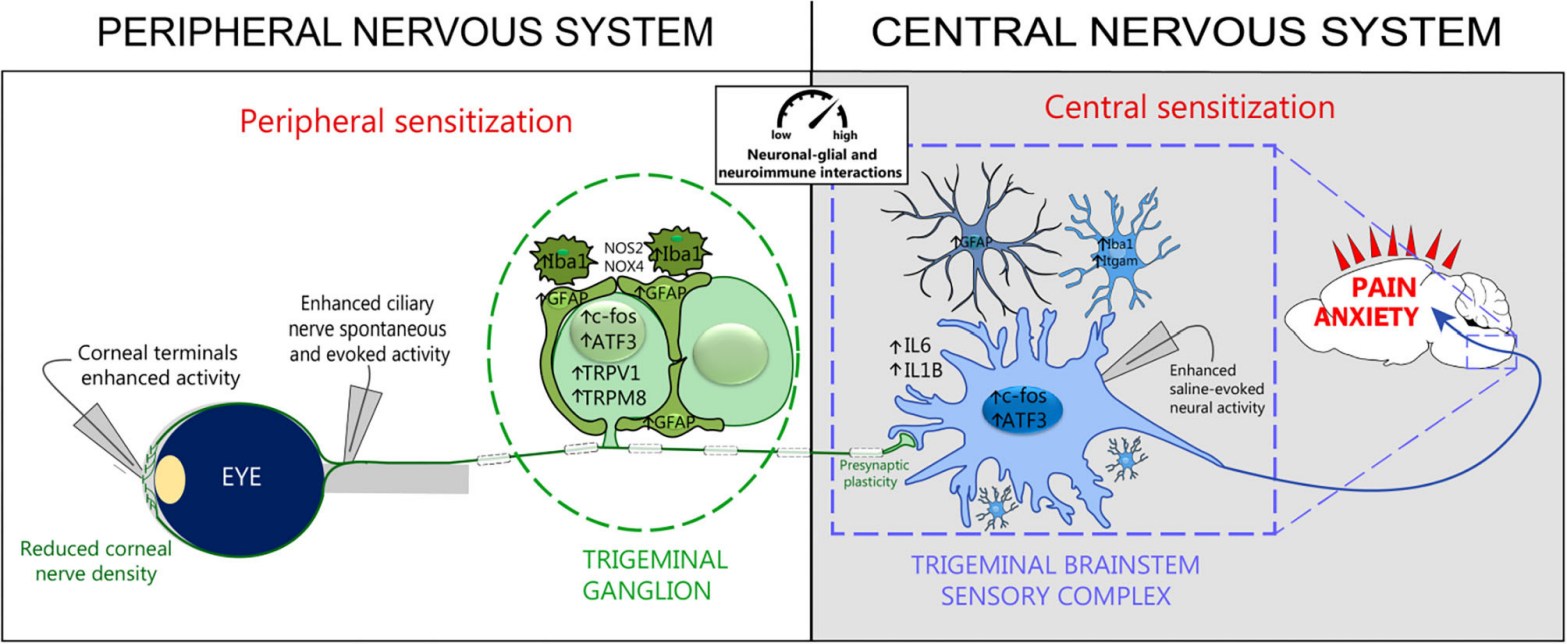
Peripheral and central sensitization associated with persistent dry eye pain. Experimental persistent dry eye triggers corneal nerve abnormalities, neuroimmune interactions, and peripheral and central sensitization. Electrophysiological recordings reveal increased activity in corneal terminals, ciliary nerves, and trigeminal brainstem sensory complex neurons. Histological and molecular studies show neuronal activation (c-Fos) and injury (ATF3), *de novo* channel production (TRPV1, TRPM8), glial activation (Iba1, GFAP, Itgam), oxidative stress (NOS2, NOX4), proinflammatory responses (IL6, IL1B), and synaptic-enhancer plasticity at the level of TG and TBSC. These cellular and molecular changes lead to pain and promote anxiety-like behaviors. Modified from Guerrero-Moreno et al. (2021).

Recent studies have shown that acute (Launay et al., 2016) and persistent (Fakih et al., 2019) ocular pain conditions induce a higher density of Iba1-immunopositive microglial cells and higher levels of CD68 and ITGAM genes in the ipsilateral trigeminal brainstem sensory complex. Aside from immune cell activation, increased levels of the GFAP protein and gene expression, astrocyte marker in the brain, were reported in this central structure. Moreover, the upregulation of pro-inflammatory markers IL-6 and IL-1β, INOS2 genes, and ATF3 and FOS markers in the trigeminal brainstem sensory complex (TBSC) was associated with the development of corneal hypersensitivity (Fakih et al., 2019) (Figure 4). These findings strongly suggest that neuronal–glial and neuroinflammatory interactions occur after corneal nerve injury in the central nervous system, which may account for the development and persistence of the ocular pain reported in DED patients.

Persistent ongoing activity in primary nociceptors may also lead to central sensitization, which is defined as “*an amplification of neural signaling within the central nervous system that elicits pain hypersensitivity*,” (Woolf, 2011) and a functional remodeling of presynaptic sites. Piccolo, one of the components of the presynaptic zone, plays a key role in synaptic plasticity by facilitating/managing the secretion of synaptic vesicles, and increased expression was reported in an orofacial pain model (Thibault et al., 2016). The upregulated expression of Piccolo in the brainstem in mice with persistent ocular pain associated with DED indicated that persistent ongoing corneal nociceptor activity induced profound synaptic reorganization, which may contribute to the chronicity of ocular pain. These central cellular rearrangements (neuroinflammation, astrocytes and microglial activation, and enhancements of excitatory synaptic transmission protein) are in line with the reported sensitization of ocular-responsive neurons of the caudal trigeminal brainstem in persistent tear deficiency in rats (Rahman et al., 2015) (Figure 4). These neuroplastic changes observed in the TG and TBSC under corneal pain are likely to play a role in the establishment and maintenance of central sensitization that is seen in many centralized pain disorders like fibromyalgia and migraine.

There is also a wealth of evidence to show that contralateral structural and molecular changes can occur both in the periphery and the central nervous system in response to a unilateral insult (Koltzenburg et al., 1999; Shenker et al., 2003; Ferrari et al., 2014; Lee et al., 2019).

For example, unilateral corneal damage induces inflammatory responses in contralateral mouse eyes (Yamaguchi et al., 2016; Lee et al., 2019), the TG (Ferrari et al., 2014), and the central nervous system (Launay et al., 2016). Indeed, FOS-like positive neurons were seen in the contralateral trigeminal brainstem after corneal nerve damage (Launay et al., 2016). Contralateral responses were demonstrable not only in rodents, but also in the human eye (Hamrah et al., 2010, 2013; Postole et al., 2016; Yamaguchi et al., 2016), and there are several reasons supporting that these contralateral responses are mediated through neural mechanisms rather than reflecting a systemic effect (Shenker et al., 2003; Gong et al., 2020).

Increased corneal peripheral nociceptive input may result in neuronal activity within the higher-order neurons within the brain, particularly in regions associated with the pain matrix (the thalamus, insula, anterior cingulate cortex, prefrontal cortex, and somatosensory cortex). Only one study has been reported to date in this area of investigation. It showed in a mouse model of corneal alkali burn that corneal spontaneous pain activated the neuropathic central pain matrix (Xiang et al., 2017). Increased phospho-ERK positive neurons were detected in the subnucleus caudalis/upper cervical cord (Vc/V1), the insular cortex, the anterior cingulated cortex, and the rostroventral medulla. Many experiments are still needed to precisely determine the nature of central structures that are recruited during acute and persistent ocular pain.

## Clinical Evidence of Corneal Nerve Abnormalities and Dysfunctions

As previously stated, the new definition of DED includes somatosensory abnormalities as a core mechanism (Craig et al., 2017). The measurement of corneal sensitivity in patients could provide the first evidence for somatosensory abnormalities, but practical tools are still lacking to assess hypersensitivity and hyperexcitability, as well as explore other stimuli than simple mechanical responses. The persistence of ocular pain in a subset of patients with dry eye syndromes is a major challenge in the management of ocular pain (Rosenthal and Borsook, 2016; Mehra et al., 2020). It is therefore crucial to identify diagnostic modalities that can accurately predict or identify neuropathic pain.

Corneal sensitivity can be assessed by the Belmonte non-contact gas and the Cochet-Bonnet esthesiometers. The Belmonte non-contact gas esthesiometer allows one to measure corneal sensory abnormalities following mechanical, thermal, and chemical corneal stimulations, i.e., the detection of polymodal function for both A delta and C fibers.

Patients with dry eye exhibit corneal hypoesthesia after mechanical, thermal, and chemical stimulation, and this condition seems to be related to damage to the corneal sensory innervation (Bourcier et al., 2005). Some studies in patients with dry eye or neuropathic pain symptoms have found reduced corneal sensitivity to mechanical, thermal, and chemical stimuli compared to controls (Bourcier et al., 2005), while others have found increased mechanical sensitivity (Spierer et al., 2016). Unfortunately, complex sensing esthesiometers are only scarcely available and are not routine devices, despite their high potential for exploring DED and corneal sensitivity.

The Cochet-Bonnet contact esthesiometer is another device that has been widely used to characterize somatosensory disturbances within the human eye and more specifically for evaluations of mechanical nociceptor (A delta fibers) responses. Some studies have reported a reduced sensitivity to mechanical stimuli in DE patients (Adatia et al., 2004; Labbe et al., 2012) exhibiting a decreased corneal nerve density.

The maladaptive processes responsible for corneal pain can take place in either the peripheral or the central nervous system. Functional somatosensory testing, also known as the proparacaine challenge test, is useful for assessing the peripheral or central location of pain generators in patients. This functional test consists of a topical instillation of 0.5% proparacaine (an anesthetic commonly used in ophthalmology) and determines whether pain is abolished or not. The persistence of ocular pain suggests a central location, whereas a peripheral origin is confirmed by pain relief (Goyal and Hamrah, 2016; Crane et al., 2017). Patients with peripheral neuropathic pain would be the principal beneficiaries of topical painkillers, while systemic approaches would be preferred for patients with central neuropathic pain.

**Figure 5:**
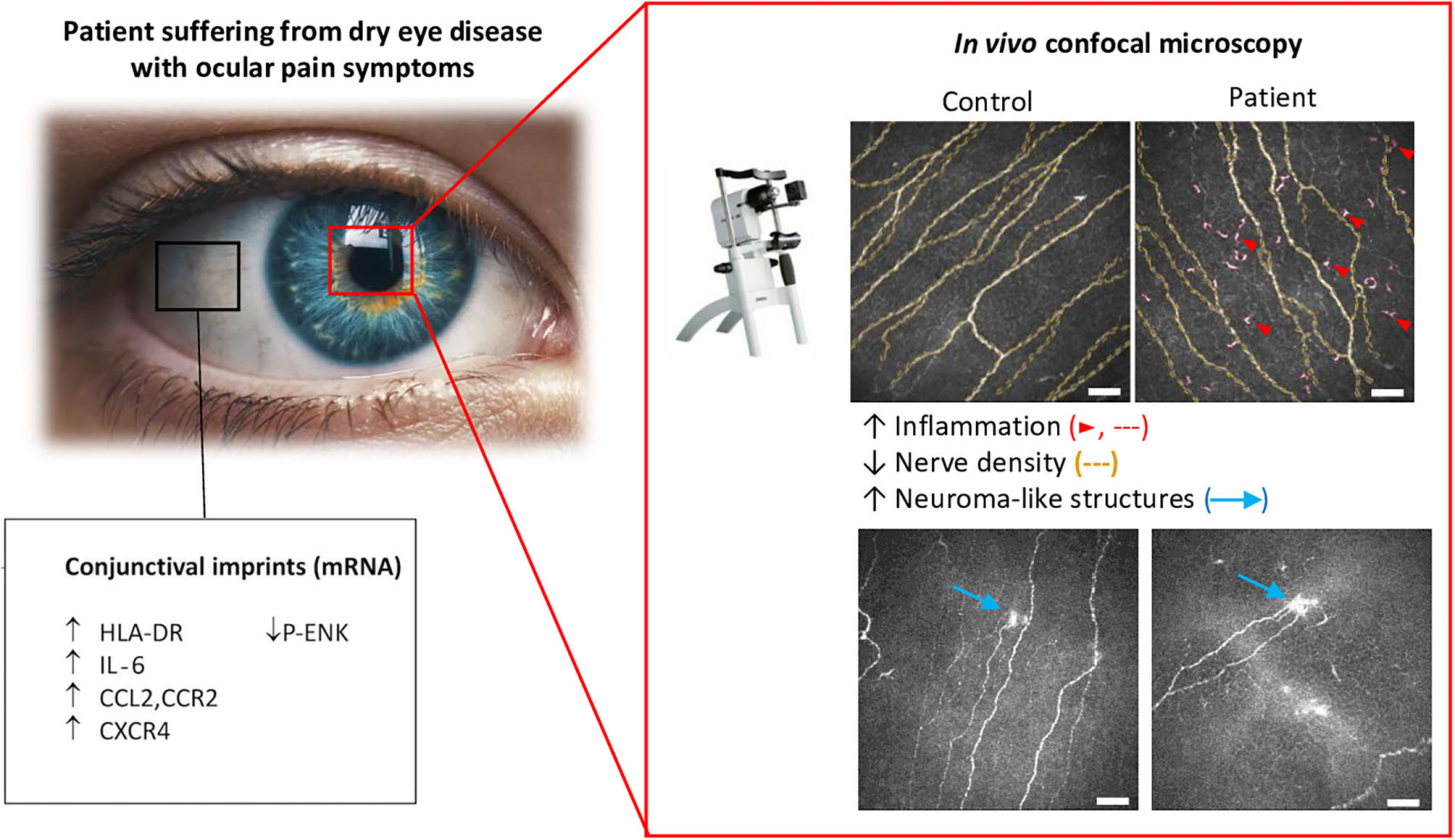
Morphological and molecular changes in the ocular surface from dry eye patients with persistent ocular pain. Conjunctival imprints show higher levels of the proinflammatory markers, HLA-DR, IL-6, and CCL2, and of the chemokine receptors, CCR2 and CXCR4. In contrast, a decrease in the enkephalin precursor P-ENK was found in DE patients. Corneal IVCM imaging showed a decrease in nerve density, which was accompanied by a higher density of hyperreflective (putative inflammatory) cells and the presence of microneuroma-like structures in DED patients with pain syndromes. Modified from Nicolle et al. (2018).

A functional assessment of corneal sensitivity is generally combined with a morphological analysis of the corneal nerve. IVCM is a non-invasive technique providing real-time, high-resolution corneal imaging in both humans and laboratory animals. IVCM studies allow for the longitudinal imaging and quantification of cellular changes in, e.g., dendritic and keratocyte cells, and of sub-basal nerve plexus morphology in corneas over time. Excellent reviews have described the broad changes in the sub-basal nerve layer by using IVCM imaging in healthy controls and patients (Cruzat et al., 2017; Al-Aqaba et al., 2019; Labetoulle et al., 2019). Quantification of IVCM images have evidenced that sub-basal nerves undergo a significant decrease in the number and density in patients with dry eye symptoms compared to healthy subjects (Bourcier et al., 2005; Benitez-Del-Castillo et al., 2007; Labbe et al., 2012, 2013; Levy et al., 2017; Nicolle et al., 2018) (Figure 5). Other morphological corneal nerve abnormalities have been observed in DE patients, such as nerve sprouting, increased thickness, tortuosity, and beading (for review Galor et al., 2018). Recent studies in patients with neuropathic corneal pain have also demonstrated decreased corneal nerve density associated with allodynia (Hamrah et al., 2017), photoallodynia (Aggarwal et al., 2015), and post-LASIK neuralgia (Theophanous et al., 2015). Those corneal nerve changes can be quantified automatically by emerging specialized software, representing a very promising tool for clinical assessments (Annunziata et al., 2016; Giannaccare et al., 2019).

Chronic pain induced after nerve injury is generally associated with prominent morphological and epigenetic changes within the afferent fibers. After nerve damage, destroyed peripheral nerves start to regenerate and form neuromas that exhibit abnormal responsiveness and spontaneous discharges (ectopic firing) (Fawcett and Keynes, 1990). These functional changes are the consequence of altered expressions of ion channel proteins in the soma and in regenerating nerve terminals. Neuroma structures have emerged as other important morphological changes in corneal nerves and have been suggested as clinical imagining biomarkers (hallmark) of corneal neuropathic pain (Aggarwal et al., 2019; Bayraktutar et al., 2020; Moein et al., 2020). Taken together, these studies suggest that a corneal neuroma may represent an important pathological feature of peripheral nerve abnormalities in patients with ocular pain. Interestingly, topical treatment with autologous serum tears reduced corneal nerve abnormalities, improved corneal nerve regeneration, and alleviated corneal pain (Aggarwal et al., 2019). Future directions are needed to determine when and how the morphological changes occur on the ocular surface and which subpopulations of corneal neurons are hit.

In recent years, clinical research regarding neuroimmune crosstalk has focused increasingly on the neuroimmune interactions in the cornea. Among the resident immune cells, epithelial dendritic cells predominantly reside in the basal epithelium of the human cornea. These resident corneal dendritic cells play a key role in maintaining the homeostasis of corneal nerves (Gao et al., 2016). However, corneal damage or corneal nerve abnormalities can disrupt the balance between immune cells and peripheral nerves. Accumulative evidence has revealed, in the cornea from patients with DED and corneal neuropathic pain, increased activated dendritic cells and a close anatomical proximity between peripheral nerves and immune cells (Shetty et al., 2016; Tepelus et al., 2017; Aggarwal et al., 2020).

As stated before, fundamental and clinical studies have well-demonstrated that repeated damage to the ocular surface and corneal nerves *per se* can cause peripheral and central sensitization mechanisms, explaining the centralized pain in some patients with corneal neuropathic pain. However, the neuronal circuits participating in corneal pain are not fully understood. Over the past decade, brain imaging investigations have shed light on neural correlations with pain perception and modulation. New developments in structural, functional, and neurochemical imaging, such as resting-state connectivity, functional magnetic resonance imaging (fMRI), and γ-aminobutyric acid spectroscopy, have shed light on persistent non-ocular pain (Grachev et al., 2000; Harris and Clauw, 2012; Legarreta et al., 2020).

To date, only one case study from Moulton et al. (2012) has reported by means of fMRI the nature of hemodynamic responses evoked by corneal pain in the human brain. fMRI imaging revealed an activation of the contralateral somatosensory cortex, ventral posteromedial thalamus, and the anterior cingulated cortex during the painful state.

## Concluding Remarks and Future Perspectives

The cornea, the most densely innervated tissue in the human body, offers multiple advantages in fundamental and clinical research, as it is an excellent model to study the function and morphology of nociceptive nerve fibers, peripheral nerve damage, nerve regeneration, and sensory abnormalities. Accumulative clinical and animal model studies have identified morphological and functional abnormalities of corneal nerve terminals associated with ocular surface diseases. The last decade has also seen a rapid expansion in our knowledge regarding the nature of the cellular and molecular mechanisms, as well as the neuroimmune interactions that take place in the cornea, the TG, which contains the primary sensory neurons, and higher central structures. However, many questions remain unanswered. Among them, unraveling neuroimmune crosstalk mechanisms leading to inflammation and pain would bring a more comprehensive picture of the entire neuroinflammatory process and represents an exciting and expanding research domain. In the future, there is also an urgent need to elucidate the time window for restoring corneal nerve morphological and functional abnormalities and to identify a more specific target and their signaling pathways in corneal nociceptors, which may offer alternative treatments.

Finally, recent advances in *in vivo* functional imaging technology, together with automatic frameworks for clinical assessment, bi- and three-photo microscopy imaging, viral-mediated optogenetic control of peripheral nerve expressing light sensitive opsins, chemogenetics, and the development of simple or dual knockout mice, may help in solving the puzzle of these maladaptive changes in the corneal nociceptive pathways.

## Author Contributions

AG-M and AR-L equally contributed to drafting the manuscript and the figures. AR-L conceptualized the review. AG-M and AR-L wrote the manuscript. AG-M, AR-L, CB, and SMP contributed to the article and approved the manuscript.

## Conflict of Interest

The authors declare that the research was conducted in the absence of any commercial or financial relationships that could be construed as a potential conflict of interest.
